# Motivation Regulation among Black Women Triathletes

**DOI:** 10.3390/sports7090208

**Published:** 2019-09-10

**Authors:** Candace S. Brown

**Affiliations:** 1 Department of Public Health Sciences, University of North Carolina, Charlotte, NC 28223, USA; cbrow342@uncc.edu or; 2 Motivated Cognition and Aging Brain Lab, Center for Cognitive Neuroscience, Duke University, Box 90999 Durham, NC 27708, USA

**Keywords:** motivation, triathletes, regulation, Black women

## Abstract

There is a paucity of information on motivation among U.S. minority triathletes. This study aimed to understand the extrinsic motivation and regulators of Black women triathletes using a modified version of the valid Motivations of Marathoners Scale and semi-structured interviews, for triathletes. The Self Determination Theory guided the dual method assessment of the extrinsic motivators and the regulators external, introjection, and integrated. Using MANOVA, data from (*N* = 121) triathletes were compared across participant categories of age, body mass index, and distance. Results showed a significant age difference with younger women displaying more motivation. Descriptive means indicated integration as the greatest regulator of motivation. The statements ‘to compete with myself’ and ‘to be more fit,’ had the highest means among the women. A sub-sample of 12 interviews were conducted revealing 16 extrinsic themes. Six were related to the regulator integration and two unexpectantly related to the regulator, identified. Integrated themes, including coping mechanisms, finishing course, improvement, accomplishment, and physical awareness were most represented. This research fills gaps of understanding extrinsic motivation and the regulators of a group not previously explored. Future research on motivation among triathletes may benefit knowing how motivations are regulated, as to promote personalized training and participation.

## 1. Introduction

Motivation is simply defined as the, “direction and intensity of one’s effort,” [1] (p. 51) and is self-initiated for direction and sustainability leading toward a goal. Preparation for sport participation is intentional. Therefore, the behavioral construct of motivation is principal to understanding why people choose to participate in sport [2].

Motivation to participate in sport and understanding cultural constructs have drawn from an array of theoretical perspectives from many disciplines. The Self Determination Theory (SDT) is a macro theory of human motivation that addresses issues such as self-regulation, psychological needs, life goals and aspirations, energy and vitality, and a host of other issues related to well-being and life domains [3]. It specifically determines how behavior describes both personal choice and outside influence, therefore, allowing for an examination of the differential effects of motivation that underlie the behavior of sport participation [3,4]. However, behavior is not always intrinsically motivated and certain external pressures (e.g., social) may motivate individuals to participate in sport even though it does not interest them.

A sub-theory of the SDT, the Organismic Integration Theory (OIT), explains the external contextual processes that serve as barriers or facilitators in behavior regulation [5]. A central assumption of the OIT is that extrinsic motivation can be measured by regulators of a continuum that range from external regulation to integrated regulation [5]. This continuum of motivational regulators stimulate behavior as athletes interact with their sport environment. The four associated regulators are described through associated processes of rewards or punishments (external), due to self-control (introjected), of personal importance (identified), and having self-awareness (integrated) [3].

Research on motivation and sport reports differences in motivation based on sport level; whereas, intercollegiate and elite athletes report higher levels of motivation compared to intramural athletes and non-elite athletes [6]. Specifically, understanding motivation in the sport of triathlon provides researchers access to an extreme sport where exercise regimens can be in excess of 20 h per week as it requires practice in the three separate sports of swimming, cycling, and running [7]. Studies on triathletes reporting differences by sex report women to have a higher fitness motivation, lower mean external regulation than men [2,8]. Motivation for initially pursuing the sport and remaining in the sport has been documented. Social reasons for participating have included a ‘sense of belonging’ [9] and physical health motivation has been related to increasing or maintaining fitness [10]. Additionally, researchers have also long considered psychological health of participation in triathlons with the literature demonstrating an increase of self-esteem, self-efficacy, and the suggested transformative effect participation has on body image [9].

Most studies on motivation among triathletes have been conducted primarily with White men and women, demonstrating a paucity in research on participants of minority status in the United States. Indeed, the number of Black people who participate in the sport is significantly lower than that of their White counterparts. Data from the 2016 survey conducted by USA Triathlon (USAT) indicated only 1% of members were Black triathletes [11]. In the same year, the Centers for Disease Control and Prevention estimated that only 44.4% of U.S. Black adults met the 2008 U.S. physical activity guidelines which were to engage in at least 150 min of weekly moderate intensity aerobic activity and to complete muscle strengthening activities that work all major muscle groups at least twice weekly [12]. Black women are a most sedentary group in the United States exercise, thus contributing to the high number of those diagnosed with death-causing conditions such as coronary heart disease and cancer [13,14].

However, Black women who participate in triathlons represent a group of successful exercisers with limited exposure in research. The only known study on Black women to participate in a minority-based triathlon program (*N* = 25) reported that the top motivational reason for participation was to improve their health and fitness (84%). Furthermore, while many of the participants (48%) reported they were motivated by the group to prepare for a triathlon, receiving encouragement from friends was one of the lowest responses for motivation, at 28 percent [15]. Therefore, the purpose of this study was to explore the extrinsic motivation and regulators of Black women who participate in triathlons to further develop an understanding of motivation among triathletes.

A measure used to understand varying motivation and underlying themes for participating in sporting events and activities is the Motivations for Marathoners Scale (MOMS). Developed by Masters, Ogles, and Jolton [16], the MOMS uses four motivational categories: physical health, social, achievement and psychological to assess motivation. Within these four categorical measures (Physical Health, Achievement, Psychological and Social) of the MOMS, there are nine scales (i.e., General Health Orientation, Weight Control, Affiliation, Recognition, Competition, Personal Goal Achievement, Psychological Coping, Self-Esteem, and Life Meaning). The MOMS has been used in several studies related to athletes who participate in sport disciplines related to triathlon. Male cyclists were more motivated by Competition, and female cyclists the scale Weight Concern, as reasons for cycling [17]. Participants of cause-related (to support charity) Aquabike (i.e., swim and cycle) and cycling only events were significantly more motivated by Personal Goal Achievement (*p* < 0.001) and Competition (*p* < 0.001) compared to those who participated in non-cause-related events [18]. Triathletes registered with Triathlon Australia indicated life meaning to be the only significant variable between the elite and non-elite triathletes, (F = 4.395, *p* < 0.05), whereas elite participants felt they had more life purpose for competing in triathlons [19]. Lovett utilized a modified version of the MOMS by adding words synonymous to represent the exercises completed by triathletes (e.g., swim, bike, and run) reporting that women have greater personal goal achievement and self-esteem scores than males [20].

While these studies yielded demographic differences in the motivation among majority triathletes, what remained unclear is whether there were similar motivations among triathletes that identify as Black. The MOMS only identifies how much motivation is determined by the scales. For example, enjoyment of participating in sports has been interpreted as intrinsic motivation and competition as an extrinsic motivation, interpreted [21,22]. This study purposed to explore the motivations of Black women triathletes and to identify the regulators associated with extrinsic motivations.

A proposed link between the SDT motivations, OIT regulators and a modified version of the MOMS, intended for use with triathletes and renamed the Motivations of Marathoners Scale for Triathletes (MOMS-T). The MOMS-T was constructed to explore the motivations and regulators of Black women (BW) triathletes. Not previously done with the MOMS, the working hypothesis of this study was formed from the scale descriptions of the MOMS and from the review of qualitative descriptions of extrinsic motivation from Lamont and Kennelly [10]. The exploratory SDT/OIT/MOMS-T model would provide an understanding of how the women perceived their regulation based on their narratives. The following questions drove the analysis of the interviews:

(1) Do narratives of Black women triathletes support the placement of the MOMS-T scales weight control and competition as external regulated?
(2) Do narratives of Black women triathletes support the placement of the MOMS-T scales recognition and self-esteem as introjection regulated?
(3) Do narratives of Black women triathletes support the placement of the MOMS-T scales health orientation, personal goals, and psychological coping as integration?

It was hypothesized that external regulation would be the measured sum of the scales weight control and competition. The sum of scales recognition and self-esteem was introjection. Integration regulation was measured as a sum of scales health orientation, personal goals, and psychological coping.

The objective of this exploratory study was to understand the regulations of the extrinsic motivations, among BW triathletes. This information may inform future research using the MOMS-T and the development of programming for women interested in increasing their physical activity, and possibly completing a triathlon.

## 2. Materials and Methods

A dual method of collecting both quantitative and qualitative data guided this study [2]. The quantitative goals of the study were to use the previously validated Motivations of Marathoners Scale for Triathletes (MOMS-T) to assess extrinsic motivation for triathlon participation among BW triathletes and identify whether rates of extrinsic regulators varied by age, body mass index (BMI), and triathlon distance. The quantitative approach was non-experimental with a correlational design that was used to administer a web-based survey. The primary qualitative goal was to gain a better descriptive understanding of the relationship between extrinsic motivation and the regulators. The qualitative approach was completed through semi-structured interviews with a subset of the BW triathletes who had recently completed the web-based survey. Intrinsic motivation and their regulators are not reported here because they are less likely to explain how controlled factors effect motivation. The results reported here are based on combined data of the extrinsic motivations of the sample.

### 2.1. Procedures

Following approval from the Virginia Commonwealth University (VCU) Institutional Review Board, BW triathletes were recruited for participation in the study. Inclusion criteria for participation included self-identifying as a Black woman, age ≥ 36 years, U.S. resident, and either completion of an individual triathlon between the years 2012 and 2014 or preparing for an individual triathlon in 2015. Potential participants were directed to a web link by VCU’s secure web-based system (RedCap) that included informed consent forms, a demographic questionnaire, and the MOMS-T survey. Following consent and completion of all forms related to the quantitative phase of the study, the women were invited to participate in a semi-structured interview to further explore their motivations that were identified in the survey. Interviewed participants signed a separate consent form and agreed to the use of pseudonyms for confidentiality. Audio recording and field notes were used to keep an accurate account of the conversation between the interviewer and participants. In addition, field notes assisted in recording of follow-up questions, included descriptions of non-verbal communication, and reflected notes on the participants’ experiences. The study survey and interviews were conducted for 12 weeks, from February to May 2015.

### 2.2. Population and Sampling

Multiple sampling strategies were used for this study. Snowball sampling was used to identify Black triathlete women for study participation. Early sampled participants (*N* = 320) were sent emails from the national triathlon organization, USA Triathlon (USAT). In addition, multiple triathlete social networks, including The Black Triathletes Association, Sisters Tri-ing, the International Association of Black Triathletes, Sole Tri-Sisters, and Realizing Your Potential Everyday, posted the study information on their Facebook^©^ pages. Early participants were asked to identify other triathletes who met eligibility criteria to participate in the study. Many of the participants did this by ‘tagging’ other women on the Facebook^©^ social media pages where the original study information had been posted.

Purposive stratified sampling was used to select interviewees. Selection was based on age, estimated BMI, and distance of most completed triathlons. Those who completed the survey first, and agreed to do the interview, were first considered for the interview. Projected quotas allowed for qualitative comparisons among 12 participants with nine varying attributes. Quotas were set to represent an equal number of participants from each stratification variable. To achieve further sample representativeness, participants were from different USAT regions and states. Those who completed the surveys first, were willing to be interviewed, and met the necessary criterion of the remaining projected quotas were contacted first for interviews. Potential interviewees were first emailed and follow-up conversations through either email or telephone followed to set up the interview.

### 2.3. Instruments in the Study

Three instruments were used for this study to quantitatively and qualitatively assess the triathletes. The descriptive demographic and triathlon participation information was assessed via the Motivation of Triathlon Participation Questionnaire. The MOMS-T characterized motivation for participation in triathlons and the Motivations of Marathoners Scale for Triathletes Interview Guide (MOTIG) inquired additional information related to the motivations in the MOMS-T [13].

The Motivations of Triathlon Participation Questionnaire comprised 16 items (open and closed) related to the demographics (e.g., educational status, relationship status), triathlon participation, and current training regimen. Age was separated into two categories with ages 36–49 representing middle age and those 50+ and older representing the older age group. Four BMI categories, including underweight, normal weight, overweight, and obese, were calculated from participant self-reports of weight and height [23]. The distances and number of triathlons completed were self-reported as either Sprint, Olympic, Half-Ironman, or Ironman [24].

The MOMS-T is a revised form of the valid and reliable Motivations of Marathoners Scale in which four of the 56 statements included the exercises ‘cycling and swimming’ to replace ‘running’ and the word ‘triathlete(s)’ replaced ‘runner(s)’ to assesses motivation to participate in triathlons [13,16]. A similar form for triathletes, previously validated, involves a 7-point Likert scale to assess the importance of participation, where the number 1 indicates that the statement is “not a reason” and the number 7 indicates the statement is a “very important” reason for participation [20]. The score on each MOMS-T scale is the mean of the survey items that compose that scale.

The previously published Motivations of Triathletes Interview Guide (MOTIG) is an exploratory instrument developed specifically to complement the questions in the MOMS-T survey [13]. Its purpose was to encourage an in-depth dialogue that would reveal the thoughts, attitudes, and opinions about motivation to participate in triathlons through a newly developed method of ‘Survey Transformation’. In addition, the MOTIG inquired further to identify specific regulators of the extrinsic scales of the MOMS-T. The MOTIG facilitated discussion with participants by asking them to retrospectively consider personal perceptions from their recently completed MOMS-T survey. Corresponding statements from the MOMS-T were rewritten with an introductory clause related to the MOMS-T scale and followed by an item from the scale. For example, to transform the MOMS-T Psychological Coping scale, interviewees were prompted with the statement, “My motivation is connected to my coping ability so I am able…”, and an item from the MOMS-T scale followed, ‘To concentrate on my thoughts.’ Two to three follow-up prompts to facilitate discussion were also used for each of the 27 open-ended MOTIG items. The semi-structured format allowed for flexibility and exploration of new concepts and themes.

### 2.4. Converging the SDT, OIT, and MOMS-T

Before the analysis, the conversion of SDT motivations, OIT regulators, and data from Lamont and Kennelly was used to propose which MOMS-T scales for participating in triathlons were extrinsic [10,21]. Key words describing the extrinsic motivation of this study were matched with the scales within the MOMS-T. Table 1 illustrates the process that led the creation of the extrinsic MOMS-T scales to the OIT-associated regulators. External regulation, considered to be the least self-determined form of motivation, would regulate the MOMS-T scales weight control and competition. Triathletes who engaged in the sport to avoid negative feelings or reprimand from others, were *introjection* regulated through the scales self-esteem, and recognition. Those with integrated regulation chose to participate in triathlons because they had fully developed their value systems. Those values were attached with personal goal achievement, health orientation, and psychological coping. The only regulator that was not included to have a link with the MOMS-T was *identified*. This regulator is theorized to represent participation in a sport, even if the activity is unattractive [25]. It was believed that the women who participated in a triathlon would disregard any notion of the activity being ‘unattractive,’ participate if it meant their personal goals would be met and would, therefore, already identify as a triathlete.

### 2.5. Statistical Analysis

The quantitative analytic plan was to use IBM SPSS Statistics (version 22) to assess participants’ extrinsic motivation for participating in triathlons and to identify differences of the seven scales and three regulators. Mean values of the 7-point Likert-format scale were calculated from the extrinsic MOMS-T statements (General Health Orientation, Weight Control, Recognition, Competition, Personal Goal Achievement, Psychological Coping, and Self-Esteem). Next, a new dependent variable termed “extrinsic motivation” was calculated by aggregating each individual’s mean scores across her/his responses to MOMS-T items on the extrinsic scales. MANOVA analysis would show the correlation between the dependent regulators (weight control and competition = external; recognition and self-esteem = introjection; health orientation, personal goals, and psychological coping = integration regulation) and variables age, BMI and distance.

The qualitative analysis descriptive comparisons of the most motivating and least motivating MOMS-T items for this study population were assessed. To accomplish this, thematic analysis of qualitative data was conducted to further explore the relationship between motivation and regulators identified in the MOMS-T survey by participants. The researcher based the interview questions in the questionnaire on the quantitative MOMS-T survey and developed three additional questions to understand how the women started in the sport and their plans for continuation of the sport. All participants chose pseudonyms to protect their identities. The audio recording was transcribed verbatim and the field notes were used as supplemental data. All collected data were entered into the qualitative data analysis program, Atlas ti.7 and quotations representing categories of extrinsic motivation, identified by phrases, sentences, or paragraphs, were assigned a label. The labels were then categorized and regrouped according to their similarities. Categories reflecting more than 70% of the interviews were identified as adequately saturated [26]. These categories were then collapsed into common definitions to help identify themes to explain the participants’ motivational views. Member checking and peer review of the codes and themes were conducted to promote trustworthiness of the data.

## 3. Results

### 3.1. Quantitative Results

A total of 140 people responded to the survey with 121 meeting the inclusion criteria. Nineteen were excluded: (a) three for criteria exclusion (one under criterion age and two men); (b) nine for duplicate submission, and seven for incompletion). Two of the incomplete survey participants were contacted and encouraged to complete their surveys as they had, at least, completed the demographic portion of the survey. Table 2 displays the survey participant characteristics. Among 118 participants who provided their age, most were younger (76.9%). BMI calculations from self-reported height and weight data, indicated most of the triathletes were either normal or overweight (61.2%). The data from the one participant who was calculated to be underweight were collapsed into the normal weight category. More than 90% of participants had completed a triathlon in the past 3 years, and the 8.3% of participants who had not yet completed a triathlon expected to complete a triathlon by the end of 2015. The Sprint distance triathlon category was the most completed triathlon distance among participants (48.8%).

The descriptive statistics of the seven scales which included 43 (of the 56) survey items show the possible minimum and maximum numbers indicate that health orientation, personal goal achievement, and recognition had large ranges. Psychological coping and self-esteem, with higher min and max scores, also had large ranges. When compared to the other scales, weight control and competition had low ranges; both scales only had four values. While the range of these scales varied widely, participants were more closely aligned with health orientation, personal goal achievement, and recognition. The statistics are presented in Appendix A.

Means and standard deviations were calculated for the motivational statements and presented in Table 3. The highest rated extrinsic motivational statements were ‘compete [with] self’ (M = 6.1, SD = 1.1) within Personal Goal Achievement and ‘[to be] more fit,’ within Health Orientation (M = 6.1, SD = 1.2). The lowest rated extrinsic motivational statements were ‘beat new person’ was (M = 2.0, SD = 1.5) for the scale Competition and ‘compliments from others’ (M = 2.2, SD = 1.5) for Recognition.

The descriptive statistics of the extrinsic regulation variables (aggregated by responses to the extrinsic scales) and categories of age, BMI, and distance indicated integration regulation was the highest, and external regulation was the lowest, in all categories. Older participants (50+) reported more integration regulation than the younger participants did, those with normal BMI strata reported less integration regulation compared to the participants who were obese (which was only slightly more than those who were overweight), and the triathletes who participated in Ironman distances displayed more integration regulation than that of the triathletes preferring the three shorter triathlon distances. The table is presented in Appendix B.

Results of MANOVA indicated a significant difference between the two age categories (Wilks’Λ = 0.068, *p* > 0.05). The mean for the younger women (36–49 years old) was higher than for the older women (50+ years old). Next, two-way MANOVA analysis indicated the dependent regulator variables External, Introjection, Integration and Intrinsic, (which is not reported here) were only correlated with age and distance (Wilks’Λ = 0.01, *p* > 0.05). The age and distance model of predicted means with confidence intervals, ndicated that the integration, was the regulator of motivation for this population.

### 3.2. Qualitative Results

Interview results are based on a purposive sample (*N* = 12) of participants’ perceptions and interpretations as triathletes and as Black women. Eligibility for an interview was contingent on completing the demographics and MOMS-T survey. Of the 121 survey participants, 118 (97%) were eligible. Eleven face-to-face interviews were conducted in the U.S. states of North Carolina, Virginia, Georgia, Illinois, Michigan, Colorado and Massachusetts. These states represented the Mideast, Southeast, North Central, and Northeast USA Triathlon regions. One telephone interview was conducted with a participant from California, of the West region. Presented in Appendix C are the characteristics of the interview participants (using pseudonyms), including six participants in each age group (36–49; 50+), four Sprint, two Olympic, three Half-Ironman, and three Ironman finishers; and four of the participants had a normal BMI, five were overweight, and three were obese.

There were 508 quotations related to extrinsic motivation. These quotations were coded and were separated among the original seven extrinsic scales. As set in the proposal, the researcher identified categories that were adequately saturated, reflecting more that 70% quotations. The scales within the MOMS are not as easily defined into a regulated style simply based on the scale itself. Rather, the themes within the styles are what mitigate whether a scale is external, introjection, integration, or identification.

**Figure 1:**
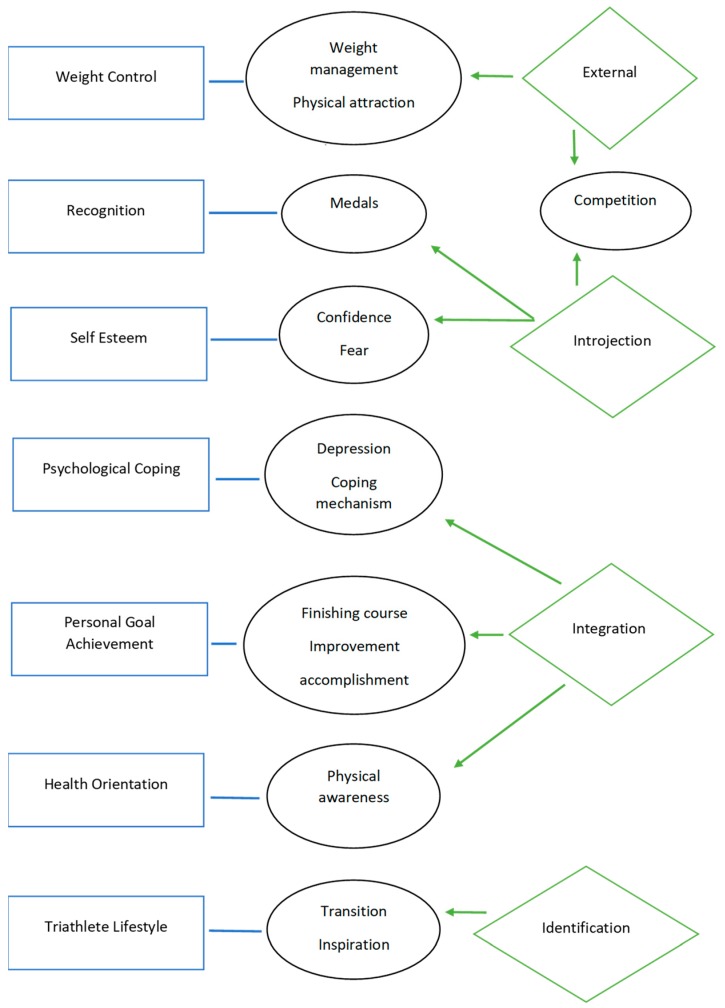
The relationship between the MOMS-T Scales, themes, and their OIT Regulators. On the **left** (blue rectangles) are the MOMS-T scales. In the **middle** (black circles), are the themes derived from the qualitative interviews. To the **right** (green diamonds), are the OIT extrinsic regulators.

There were 16 total themes gleaned from the qualitative data. Eleven themes, including weight maintenance, physical attraction, competition, medals, confidence, fear, competition, coping mechanism, finishing course, improvement, accomplishment, and physical awareness were directly related to the extrinsic MOMS-T scales and regulators. The theme—depression—is also included as the saturation was close at 69%. Transition and inspiration were themes related to the not-previously included regulator, identification, and to the new proposed MOMS-T scale, Triathlete Lifestyle. Two themes, encouragement and family, are not presented in this manuscript because of their dual relationship with the intrinsic scale, Affiliation. Figure 1 represents the total of seven scales, four extrinsic regulators, and 14 themes from the qualitative data. This section presents an analysis of the interviews and is based on the generalization of what the researcher understood about participants’ personal values and beliefs. The researcher examined whether the participant’s views about the MOMS-T motivations were supported by the intended regulators.

**External regulated.** The SDT scales, weight control and competition, were initially thought to be external regulated. Signified by the themes, weight maintenance and physical attractiveness, women were either ordered by physicians or encouraged themselves to lose weight due to medical issues. The desired effect of weight loss was to have a more defined ‘lean’ look of one who is athletic. Being lean was perceived to be more physically attractive and was evaluated based on mirror appearance or achieving the status of a ‘triathlete’ (one who completed a triathlon).

Rado explained that being physically attractive is “[being] as fit as you’re genetically able to be.” Competition, however, presented factors of being external and integration regulated. This depended on the context of the competition. The external regulation of competition occurred when a triathlete made the conscious choice to rival against teammates or other triathletes in a race. The women who had strategies for overcoming another triathlete enjoyed and often giggled at their thoughts of how they would view the other triathletes during a race. For Brooklyn Diva, it was a production where she was the hero going after the villain, entitled ‘Superhero’. “I go into a different mindset like I’m in a movie. I’m following this person. I gotta keep up and I’m just thinking the entire time, ‘You gotta catch them,’ and I just play a movie in my head.” Competition also contained an element of integration of setting a goal and essentially creating a personal and internal competition. Nita believed that, “The only person that I’m competitive against, is myself.”

**Introjection regulated.** The researcher postulated the SDT scales recognition and self-esteem to be regulated through introjection. Most participants stated they were not concerned about what others thought of their participation in triathlons and did not participate for approval. All but one participant said recognition did not directly motivate them; yet, they all appreciated receiving recognition in the form of encouragement, compliments, respect, or motivation for other women to participate. Achieving different feats within the sport is cause for celebration but the level of celebration is not defined by the distance of the race. For example, the new sprinter who completed her first triathlon spoke of how much recognition she received just like the three-time Ironman finisher did. Their eyes lit up with such pride and a smile cut across their faces, as though they could recall every encouraging word they received during training.

Medals (i.e., bling) were a form of recognition that were regulated as introjection. The medals serve as ‘proof’ that the triathletes had completed a course and were most often displayed by the participants. They also serve as a reminder of the participants’ past experiences and accomplishment. In this way, medals are also be described as external regulated because some triathletes would not participate in certain races if they knew they would not receive, at least, a finisher’s medal. Hanna, explicitly said, “I do it for the bling—but that’s about it.”

The MOMS-T scale, self-esteem, was marked by two themes, confidence and fear. Confidence can change over time and is built based on experience. The women who had not been racing as long expressed that participating increased their confidence. The older women expressed that they overcame self-esteem issues years earlier so now self-esteem is, “like the cherry… it’s like that added little benefit,” said Melle.

Fear, a theme which described a mental to physical paralysis, could occur while training or during a race if it was not overcome. While there was some type of fear in all three disciplines, fear of swimming was discussed the most. There were levels of fear of the swimming including: (1) fearing the pool; (2) fearing the deep end of the pool; (3) fearing the fresh open water; (4) fearing the animals (i.e., fish) that might be in the fresh open water; (5) fearing the saltwater; (6) fearing the bigger animals that might be in the saltwater (e.g., alligators). The two fears in the cycling portion of triathlon were falling off the bike or getting hit by a vehicle while riding. The fear of getting hit by a vehicle was synonymous with running too, as well as not being fast enough to get away from a dog chase. The location of the dog did not matter either because dogs live everywhere—in the city and the country. Recognition and self-esteem were not one-dimensional regulated scales due to the differences in the how the themes were regulated.

**Integration regulated.** The researcher speculated integration to regulate the SDT scales of personal goal achievement, health orientation, and psychological coping. Every one of the participants found personal goals important as there was a sense of pride with setting goals and achieving them. The themes related to personal goal achievement had equal weight. It was motivating to finish the course, no matter the obstacles. Additionally, it was equally important to improve in either one’s personal time in one or all the disciplines, or in one’s finish time. Draya expressed, “What’s … important is to… see if I can beat the time I had before on that same course.” There is a difference between the personal goal achievement themes of finishing the course and accomplishment. Accomplishment occurred after completing the race and the feeling was reported to carry into other parts of life. Finishing the course simply meant they crossed the finish line; however, crossing the line did not have the overflow into other parts of life.

The theme of physical awareness represented the scale, health orientation. The process of awareness was personal and for an athlete’s health to be integrated, a synthesis of meaningful aspects related to triathlon occurred. Experiences made the older triathletes realize how necessary having good health is. Awareness was described as being integrated into their motive for training; once training started, they could experience the effects physically, spiritually, socially and psychologically.

Psychological coping was also regulated through integration as triathlons were described as a coping mechanism to get through life by the majority of the participants. They reported using the physical aspect (i.e., health orientation) to cope with anger and the stressors of life. One of the three disciplines was a favorite or the ‘go-to’ form of exercise for the women. Eve stated, “Whenever I’m upset or angry, I take it out on the pavement.” In addition, Marissa jokingly expressed that, “This world is a safer place because I’m doing triathlons. I just need to tell you that right now, there are people who are walking around alive today because I’m doing triathlons.” Similar to what was experienced with fear, the theme depression was either a barrier or served as a motivator to complete goals. However, depression was more integrated because it directly regulated behavior (rather than being an external regulator like fear). Several of the women used triathlon to free themselves from negative behavior caused by their diagnosis of depression. Elle was the first to explain, “I do have depression but [when] taking medication… I just didn’t like the side effects… so, I just did my research. Exercising was the thing that made me feel good.”

**Identified regulated.** Identified is the final form of extrinsic regulators which the researcher had not previously thought to be a regulator among the SDT scales. Two themes, inspiration (under recognition) and transitions were analyzed to be identified regulators attached to a new proposed scale Triathlon lifestyle. The women spoke of how they were inspired to be triathletes and then recognized their own ability to be an inspiration. However, in order to maintain the ability to inspire others, the participants adopted a sense of accountability. Nora stated, “I just want to be an example to others, in terms of being in good physical shape and looking the part.” While they experienced their autonomy of continuing to practice the sport, it became a practice that engaged more women to participate, thus benefitting others as well.

The theme, transition, was the self-identification of owning the title ‘triathlete.’ This was not as easily adopted by some of the women because of the more socially accepted and described views of what a triathlete should look like. However, as the women furthered their pursuit of adopting the lifestyle of a triathlete, they also came to identify themselves as a triathlete—regardless of their personal looks.

**Triathlon lifestyle.** The new theme of triathlon lifestyle emerged from the interviews. It is described as a ‘motivation that is or is perceived to be sustainable over time, as opposed to motivation that is temporal’. The lifestyle, itself, is a journey one takes when they begin the sport, continues through participation in the sport and looks forward toward aspirations for the sport in later life.

When beginning the sport, there are those who do are ‘one and done’. This is a person who has likely checked off ‘triathlon’ from their bucket list. However, a person who ‘catches the bug’ has the potential to become a junkie who is addicted to the lifestyle. The lifestyle triathlete, however, recognizes that after they have spent weeks or months to prepare for a race, they must continue to live this lifestyle because completing one race is not enough. To continue to be successful, the triathlete’s preparation becomes a lifestyle. Some of the women of this study thought triathlon would be a onetime event in their lives, too, but they felt differently after their first experience. For Letti, the transition to recognizing her lifestyle change occurred when she came to respect what triathlon did for her beyond the physical. “When I first started…I did not really appreciate – did not recognize the healing of stress, of problem solving. It does so much for you. But, at the beginning? I didn’t recognize that.” The usage of the words being ‘hooked’ or ‘junkie’ and ‘crazy’ became descriptor words the women heard from others and proudly adopted for themselves. Lexi recognized that making triathlon a lifestyle and being part of the triathlon family is fine because it was, “…encouraging that I’m not [the only] crazy person. Here’s a group of people that have a similar goal and so it’s been encouraging me to continue and work on that. When everybody is crazy, nobody is.”

## 4. Discussion

Motivation to participate in triathlons is gaining exploration, lending to a variety of motivation constructs that vary across populations. This study adds to the knowledge of diversity of triathletes by including a group of minority women. Using dual methods, the motivations and regulators of triathlon participation were examined among 121 Black women, aged 36 and older. The theoretical focus on extrinsic motivation was characterized by Self Determination Theory (SDT), a theory of human motivation that addresses issues such as psychological needs, goals, and other issues related to well-being [3]. The sub-theory of SDT, the Organismic Integration Theory (OIT), defines those barriers or facilitators in behavior regulation [5]. To measure motivation, the researcher used the Motivations of Marathoners Scale for Triathletes (MOMS-T) as a survey to collect the motivational thought and then developed questions from the MOMS-T for semi-structured interviews which explored participants’ motivational thought for descriptive and narrative data.

Using the SDT keywords and OIT regulators to identify how the scales in the MOMS-T are regulated is novel. Other surveys, like the Sport Motivation Scale II, have also used the SDT to understand the regulators of sport motivation [2]. Unlike the SMS II, the MOMS-T did not have statements hypothesized to be identified. In the SMS II, ‘develop myself’ is a phrase consistently used with statements related to the identified regulator. The idea of personal development, in the MOMS-T, is directly related to measurable racing goals (e.g., To try to run faster) within personal goal achievement. Identified was not believed to be a regulator with this population. However, themes extracted from the MOTIG helped with the development of the new scale, Triathlete Lifestyle. This scale was also directly related to the regulator, identified. The women self-identified as triathletes because their lifestyle differed from other athletes who may only perform one of the three disciplines (e.g., marathoners) or do another form of exercise (e.g., yoga). Like the study by Armentrout, which described that Ironman triathletes go through a process of change—where they adopt a healthy lifestyle—the same holds true for all the women of this study, regardless of the distance. All transitioned into recognizing their life as a triathlete [27].

Integration was identified as the strongest SDT motivational regulator for the quantitative and qualitative methods. Quantitatively, more than 50% of the women believed it was the integration of the sport which motivated their participation. This suggests that the women’s motivations were more personal in nature and were not motivated by others’ views of their accomplishments. While the MOMS-T, alone, provided more of ‘what’ motivates a person to participate, the interviews provided ‘how’ they were regulated. The interviews revealed integration regulating four of the MOMS-T scales (Psychological Coping, Health Orientation, Personal Goal Achievement, and Triathlete Lifestyle).

Another key finding was the interpretation of the scale, Competition. The MOMS-T defines competition as competing with others and being faster than someone else. The statements were insignificant for the survey participants with an overall mean of 2.67. However, when the women were interviewed, they provided a richer explanation as to why this mean scale was low. The items did not ask about competition with themselves. Similar items that could be described as self-competition, were found under the scale Personal Goal Achievement. Statements including, ‘to push myself beyond my current limits,’ and ‘to compete with myself,’ could be considered as a form of self-competition. Yet, when the women discussed their personal goals, the themes of finishing the race, having feelings of accomplishment, is what was gleaned from the interviews—not a form of self-competition.

Using a survey of motivational factors for triathletes (i.e., MOMS-T) was a strength. Findings were comparable to previous studies on triathletes [10,28,29]. However, using the novel method of survey transformation for the dual-method approach provided additional qualitative explanations of the survey answers not previously noted. Using a secondary method of gathering data through interviews, as a follow-up to gathering survey data, was feasible for providing further understanding about certain constructs. For example, the lack of assessment of triathlon medals, as a form of external regulation, was not measured in the MOMS-T but was important to these triathletes.

Limitations to the study include methodological issues, participant representativeness, and potential misrepresentation. The sampling resulted in participant self-selection, which suggests that those who participated in this study may differ from those who did not. The surveys were only made available through computer access; it is possible that the computer sampling techniques did not reach as many subjects as possible. The researcher used convenience and snowball sampling techniques, making it difficult for the triathlon affiliated networks (e.g., USAT) to verify the legitimacy of samples’ participation rates. Due to self-reporting, the demographic survey asked questions could have been answered incorrectly (e.g., weight/height).

The results of the study lay the foundation for considering expanding the scope of analysis related to the SDT. Multiple studies, in varying disciplines, have used the SDT to understand motivation. However, self-determination has been most applied to sport, education, and health care [3,28]. Therefore, to understand how people are further motivated, the SDT regulators and how they intersect with motivation requires additional mixed-methods analysis.

One way to expand the scope of analysis of the SDT and possibly affect policy related to exercise would be to use of the Short Form (SF-12) Health Survey in additional studies. The SF-12 has most recently been used to evaluate the validity and reliability of a questionnaire developed to measure the influence of competitive sport participation on lifespan health and well-being [29]. The use of such an instrument in combination with surveys of exercise levels and motivation, like the MOMS-T, may help in understanding the effect of exercise on health status.

## 5. Conclusions

This study is a first to explore the motivations of Black women who participate in a sport dominated by white men in the United States. Understanding the motivations for Black women who participate in triathlons is important to the pursuit of health equity, as Black adults are the racial/ethnic group least likely to meet physical activity guidelines, and Black women are less active than Black men [30,31]. Although researchers have identified barriers of exercise for women, this information, not previously highlighted in research, may assist in identifying the factors to motivate Black women to exercise regularly [32].

The novel method of using survey transformation allowed researchers to glean previously uninterpreted information from a quantitative survey through the interview process. Previous research on motivation has not used a qualitative version of the MOMS; therefore, the interpretation of competition in previous studies, or other surveys like the SMS II, were not able to identify competition as a personal motivation but rather, as a motivation based on rivalry in sport. This is useful knowledge when considering previous research revealed that young girls, who abandon the sport, may not if they receive support through change of the rules of competition [33].

The interviews also confirmed that the MOMS-T survey should be revised and revalidated after including of questions relevant to the newly emerged ‘Triathlon Lifestyle’ scale. Not previously seen in studies related to motivation using the MOMS, this scale can provide a separate dimension of understanding motivation from the perspective of it being a sport maintained throughout life. Additionally, the interviews of this study also supported earlier research that stated expanding analysis to the MOMS-T by measuring the fun or enjoyment as motivation for participating triathlons [20].

The survey transformation method confirmed an adaptable approach to using surveys, in future settings, to other populations when there are insufficient resources to redo formative qualitative work that informed the initial survey. Triathlon programs, like those which have been implemented into school physical education programs [34], can be designed to motivate minorities to participate in triathlon races and may find it useful to include some motivation statements identified in this study. Future research also might seek to identify extrinsic motivations in other demographic groups, by utilizing the mixed-methods research approaches as in the present study.

## Figures and Tables

**Table 1:** Converging the Motivations of Marathoners Scale for Triathletes (MOMS-T) scales and Organismic Integration Theory (OIT) regulators.

MOMS-T Scale	MOMS-T Descriptions	SDT Keywords	OIT Regulator
General Health Orientation	Improve health, prolong life, stay physically active	Synthesis of goals	Integration
Weight Concern	Look leaner, control weight, reduce weight	External reward	External
Recognition	Earn respect, feel pride from others, earn recognition	Ego Involvement	Introjection
Competition	Compete with others, be faster than friends, placement achievement	External Rewards	External
Personal Goal Achievement	Improve speed, push myself, improve overall time	Synthesis	Integration
Psychological Coping	Be less anxious, distraction from worries, improve mood	Congruence	Integration
Self-Esteem	Improve self-esteem, improve confidence, sense of achievement	Self-esteem	Introjection

**Table 2:** Study participant characteristics.

Variable	Values	Mean	Number	Percentage
Age (years)	36–49	-	93	76.9%
	50+	45.6	25	20.7%
	Missing	-	3	2.5%
BMI	Normal	-	37	30.6%
	Overweight	-	37	30.6%
	Obese	-	31	25.6%
	Missing	-	16	13.2%
Distance completed	Not completed	-	10	8.3%
	Sprint	-	59	48.8%
	Olympic	-	15	12.4%
	Half-ironman	-	17	14.0%
	Ironman	-	17	14.0%
	Missing	-	3	2.5%

**Table 3:** Extrinsic Motivational Statements.

Scale	Highest Motivational Item	N	Mean	SD	Lowest Motivational Item	N	Mean	SD
Health Orientation	more_fit	119	6.059	1.188	reduce_heartattack	120	4.500	2.268
Weight Control	leaner_look	119	4.773	1.955	reduce_weight	119	4.176	2.154
Personal Goal Achievement	compete_self	119	6.160	1.112	beat_time	119	3.933	2.170
Competition	compete_others	120	3.242	1.932	beat_new_person	117	2.034	1.531
Recognition	famfriends_proud	118	2.754	1.719	compliments_othe	120	2.167	1.525
Psychological Coping	improve_mood	119	3.807	2.001	less_depressed	119	2.798	2.048
Self-Esteem	meaning_life	120	3.708	2.047	feel_whole	118	2.737	1.874

## Appendix A

**Table A1:** Descriptive Statistics of the seven Scales.

Scales	N	Mean	SD	Min	Max
Health Orientation	115	32.03	7.975	6	42
Weight Control	118	18.17	6.597	4	28
Personal Goal Achievement	118	32.20	6.789	6	42
Competition	117	10.75	5.914	4	28
Recognition	113	14.57	7.306	6	42
Psychological Coping	112	30.15	14.33	9	63
Self-Esteem	116	22.05	10.63	7	49

## Appendix B

**Table A2:** Descriptive Statistics of Regulators by Age, BMI, and Distance.

Category	Regulator	N	Mean	SD	Min	Max
Age						
35–50	External	90	39.54	13.31	11	77
	Introjection	87	49.29	16.47	14	98
	Integration	85	92.02	24.08	21	141
50+	External	23	43.83	15.25	20	77
	Introjection	21	51.29	14.58	22	74
	Integration	20	105.70	18.32	74	147
BMI						
Normal	External	36	37.33	14.50	11	77
	Introjection	34	47.12	19.11	14	84
	Integration	31	93.10	27.26	21	147
Overweight	External	34	41.56	13.92	16	71
	Introjection	34	48.35	13.37	20	74
	Integration	33	96.00	24.35	39	141
Obese	External	31	43.19	14.50	13	77
	Introjection	28	55.39	16.34	32	98
	Integration	29	96.14	20.91	57	141
Distance completed						
Not completed	External	10	39.20	18.65	13	71
	Introjection	9	43.11	15.96	20	64
	Integration	10	90.90	25.93	57	130
Sprint	External	57	40.23	13.93	11	77
	Introjection	55	49.62	17.20	14	98
	Integration	53	93.47	24.51	21	141
Olympic	External	14	40.36	13.39	20	59
	Introjection	13	50.54	17.62	22	77
	Integration	13	92.62	17.37	66	132
Half-Ironman	External	15	35.93	10.52	16	51
	Introjection	15	47.33	13.70	26	72
	Integration	14	94.29	25.29	39	131
Ironman	External	17	45.65	12.84	30	77
	Introjection	16	56.38	11.44	30	74
	Integration	15	102.00	24.63	72	147

## Appendix C

**Table A3:** Interview Participant Characteristics.

Participant	Age	Preferred Triathlon Distance	BMI
Brooklyn Diva	45	Olympic	normal
Lexi	51	Sprint	normal
Eve	45	Half-Ironman	normal
Elle	44	Sprint	obese
Letti	56	Sprint	normal
Nita	50	Sprint	overweight
Marissa	43	Ironman	overweight
Draya	58	Olympic	overweight
Melle	54	Half-Ironman	overweight
Rado	49	Ironman	obese
Nora	60	Half-Ironman	overweight
Hanna	39	Sprint	obese
